# Purification and characterization of mutant miniPlasmin for thrombolytic therapy

**DOI:** 10.1186/1477-9560-11-2

**Published:** 2013-01-30

**Authors:** Xiaotao Lin, Yan Wang, Yanwen Zhang, Bing Huang, James J Lin, Scott J Hallock, Hong Yu, Hongwei Shao, Jing Yan, Bo Huang, Xuejun C Zhang, Wei Cao, Xueming Xu, Xinli Lin

**Affiliations:** 1 Genecopoeia Inc, 9620 Medical Center Drive #101, 20850, Rockville, MD, USA; 2 Guangzhou FulenGen Co., Ltd, Guangzhou, China; 3 Clinical Medical and Pharmaceutical College, China Medical University, Shenyang, China; 4 Novartis Institutes for Biomedical Research, Emeryville, CA, USA; 5 Winston Churchill High School, Potomac, MD, USA; 6 Department of Cardiology, School of Medicine, Zhejiang University, Hangzhou, China; 7 South Florida VA Foundation for Research, Miami, FL, USA; 8 Vascular Biology Institute, University of Miami, Miller School of Medicine, Miami, FL, USA; 9 Zhejiang Hospital, Hangzhou, China; 10 Institute of Biophysics, Chinese Academy of Sciences, Beijing, China

**Keywords:** Plasminogen (-fragments), Thrombolysis/thrombolytic agents, Deep vein thrombosis, Drug design, Drug development

## Abstract

**Background:**

Previous animal studies by us and others have indicated that catheter-administered plasmin or its des-kringle derivatives may be more appropriate alternatives to plasminogen activators for treating thrombolytic diseases, since it has a very short serum half-life and therefore does not result in hemorrhaging. We have previously produced recombinant miniPlasmin (mPlasmin) that was proven suitable for treating peripheral arterial occlusion in animal models. However, our previous results showed that non-specific cleavage at position K^698^ of mPlasmin during activation hindered the further development of this promising therapeutic candidate. In order to minimize or eliminate the non-specific cleavage problem, we performed saturation mutagenesis at the K^698^ position to develop a mutant form of mPlasmin for thrombolytic therapy.

**Methods:**

We changed K^698^ to 16 other amino acids, with preferred *E. coli* codons. Each of these mutants were expressed in *E. coli* as inclusion bodies and then refolded, purified, and subsequently characterized by detailed kinetic assays/experiments/studies which identified highly active mutants devoid of non-specific cleavage.

**Results:**

Activation studies indicated that at those conditions in which the wild type enzyme is cut at the non-specific position K^698^, the active mutants can be activated without being cleaved at this position.

**Conclusions:**

From the above results, we selected two mutants, K698Q and K698N, as our lead candidates for further thrombolytic drug developments. The selected mutants are potentially better therapeutic candidates for thrombolytic therapy.

## Background

The main goal of this study is to develop a better thrombolytic therapeutic agent for thrombosis diseases, such as deep vein thrombosis and peripheral arterial occlusion (PAO). PAO occurs when a clot blocks artery blood flow to a distant part of the body such as the legs, arms, feet, or hands. PAO is the result of peripheral arterial disease (PAD), in which atherosclerotic plaque build-up on the artery walls leads to obstructed blood flow, leading to ischemia in blood starved limbs of the body [1].

Current treatments of PAO include angioplasty, stents, and thrombolytic intervention with Activase® (tissue-type plasminogen activator, tPA) or Abbokinase® (urokinase-type plasminongen activator, uPA). Thrombolytic therapeutics are not currently approved by the FDA for PAO because they require infusions that last a day or more and are associated with high risk of serious bleeding including stroke [2]. In humans, thrombolytic therapy with tPA has been shown to cause potentially fatal intracranial hemorrhage (ICH) in approximately 1% of patients receiving it for acute myocardial infarction [3]. The patient risk for ICH is even higher at 2.9% after prolonged infusion treatment for PAO [4]. Thrombolytic intervention against PAO has advanced concurrently with significant technical advances in catheter design and delivery, permitting local drug delivery directly into the clot. However, even under these circumstances, plasminogen activator (PA) mediated clot dissolution can be slow, cumbersome, and only partially effective, requiring 1-2 days to take effect [4]. Furthermore, because the effectiveness of PAs is dependent on local plasminogen (Plg) levels, Plg depletion problems occur due to long, retracted clots and poor circulation. Consequently, this renders PA therapy only partially effective for PAO, and carries with it potential serious side effects [5]. Therefore, alternative therapeutic options that are safer and more efficient are desired. One such alternative strategy is to directly use the activated form of Plg, Plasmin (Plm), which digests fibrin *in vivo.* In practical consideration, current advances in local drug delivery make Plm or its des-kringle derivatives attractive for PAO treatment. In particular, the issue of its very short serum half-life has been addressed through catheter infusion directly at the clot site as an alternative to IV administration. At the same time, the very short half-life of Plm offers the additional advantage of decreasing circulation of the active enzyme to non-specific sites, thereby reducing the risk of hemorrhaging and ICH.

As described above, breakdown of a fibrin clot (thrombi) into soluble components depends on Plm, a serine protease that is derived from the freely circulating proenzyme Plg [6]. Plg binds to both fibrin and fibrinogen, thereby being incorporated into a clot as it is formed. *In vivo*, Plg is activated by tPA trapped in the blood clot [7]. The resulting Plm is transformed into two separate subunits interconnected by 2 disulfide bridges. The A chain of the Plm molecule consists of 5 triple-loop disulfide kringle (Kr) domains (approximately 78-80 amino acids each), while the B chain contains a “linker” region of 20 amino acids and a serine protease domain (approximately 228 amino acids) [8]. Through laboratory manipulations, 2 des-kringle variants of Plg with potential pharmacological application have been created. One of these, miniplasminogen (mPlg), consists of Kr5, the linker, and the serine protease domain. The other, microplasminogen (μPlg), consists of only the linker and serine protease domain itself. mPlg and μPlg are also activated to miniplasmin (mPlm) and microplasmin (μPlm), respectively, by digestion at the peptide bond between R^561^ and V^562^ (amino acid number adapted from reference [8]). As in Plg, activation of mPlg and μPlg by tPA, uPA , or other PAs forms two separate subunits interconnected by two disulfide bridges.

*In vitro* studies have identified several interesting functional differences between Plm, mPlm, and μPlm which may have potential clinical significance in developing a therapeutic drug. Functionally, μPlm is distinguished from mPlm and Plm by its inability to specifically bind to fibrin; μPlm lacks the fibrin binding resides in Kr1-Kr3 and Kr5 domains [9-11]. While Plm and mPlm have similar catalytic rates in digesting fibrin, μPlm is 6-fold slower than mPlm and 12-fold slower than Plm [11]. Once fibrin bound Plm dissociates from the blood clot, it becomes immediately accessible to its principal inactivator, α2-antiplasmin (α2-AP). α2-AP first binds to specific lysine residues located in Kr5 and other kringle domains before binding to the catalytic domain, inactivating Plm for a resulting plasma half-life of only 0.2 seconds [9,12,13]. Apart from the half-life issue, the desirability for pharmaceutical thrombolysis development is Plm>mPlm>μPlm because of the fibrin binding specificity and the more rapid kinetics in digesting fibrin [14].

During the preclinical drug development stage and animal testing, we observed that recombinant mPlm has better pharmacological properties than μPlm, and decided to develop mPlm as a thrombolytic therapeutic candidate. However, during process development and scale up production, we faced a non-specific cleavage problem during activation of mPlg, hindering the development of this promising drug candidate. In order to solve this problem, we designed and screened mPlm mutants to select for those that retain the desired catalytic properties, but have much reduced tendency to be cleaved non-specifically.

## Materials and methods

### Materials

Wild type mPlg was purified in-house as described [15]. QuickChange® Site-Directed Mutagenesis Kit used for site-specific mutagenesis was from Stratagene. Chromogenic substrate pGlu-Phe-Lys-pNA (S-2403) was from Chromogenix (Sweden). 4-Nitrophenyl 4-guanidinobenzoate hydrochloride (pNPGB) was from Aldrich. NUPAGE 4-12% BT GEL was from Invitrogen. Other chemicals and protein reagents such as fibrinogen and thrombin were from SIGMA/Aldrich.

### Mutagenesis design and method

For mutagenesis studies, we changed K^698^ to 16 other amino acids, with preferred *E. coli* codons, as shown in Table 1. This resulted in 16 mutant expression vectors. A QuickChange® Site-Directed Mutagenesis Kit with the primer design method was used for mutagenesis. Each isolated mutant expression plasmid was sequence verified, and expressed the same way as the wild-type mPlg.

**Table 1:** **Preferred *E. coli *** **codons of the 16 amino acid**

**Amino acid**	**Codon ( *E. coli *)**	**Amino acid**	**Codon ( *E. coli *)**
Phe/F	TTT	Leu/L	CTG
Tyr/Y	TAT	Pro/P	CCG
Try/W	TGG	Gln/Q	CAG
Ile/I	ATC	Val/V	GTT
Ser/S	AGC	Ala/A	GCG
Met/M	ATG	Asp/D	GAT
Thr/T	ACC	Glu/E	GAA
Asn/N	AAC	Gly/G	GGT

### Inclusion body expression, purification, and refolding screening

The sequence verified mutant plasmids were transformed into *E. coli* strain BL21(DE3) for expression, refolding, and purification following the same procedure as previously described [15]. Briefly, *E. coli* containing the expression plasmids were expressed in a high-density shaker flask auto-induction system [16]. The broth was then spun down and the pellet was washed extensively and put through freeze thaw cycles with lysozyme to purify the inclusion bodies. The purified inclusion bodies were dissolved in an 8 M urea buffer (8 M urea, 0.1 M Tris, 1 mM glycine, 1 mM EDTA, 10 mM β-mercaptoethanol, 10 mM dithiothreitol (DTT), 1 mM reduced glutathione (GSH), 0.1 mM oxidized glutathione (GSSG), pH 10.5 with a final concentration of 2 mg/ml). The solution was rapidly diluted into 20 volumes of 20 mM Tris, 0.2 M L-arginine, pH 10.5**.** The pH of the solution was slowly adjusted to pH 8 with 6 M HCl as described [17]. The refolded protein was then concentrated by ultrafiltration, and purified by various types of column chromatography as described [15]. The expression level of each of the mutant mPlg was essentially the same as that of the wild-type’s. For initial screening, we grew 200 ml culture for wild-type mPlg and each of the 16 mutants, yielding about 200 mg of highly purified IB for each construct.

### Activation and kinetic measurements

Kinetic measurement was performed similarly as described [15]. Briefly, the kinetic parameters of the activated mPlg wild-type and mutants were measured with a chromogenic substrate pGlu-Phe-Lys-pNA (S-2403). The refolded and purified zymogens (35.5 μM) were activated at 37°C for 10 min in a reaction mixture containing 25 mM Tris-HCl, pH 7.4, 50 mM NaCl, and 0.37 μM of staphylokinase (SAK). The active site of the activated mPlg was titrated using pNPGB as described [18]. The activated zymogens were diluted to 5.5 μM, and then 10 μl was mixed with 100 μl of 0.0625 mM, 0.125 mM, 0.25 mM, 0.5 mM, 0.75 mM, 1.0 mM, 1.5 mM, or 2.0 mM of substrate S-2403 in the assay buffer (25 mM Tris-HCl, 50 mM NaCl, pH 7.4). The generation of amidolytic activity was monitored (at 405 nm) at 37°C in 10 sec intervals for 20 min using SpectraMax 250 microplate reader (Molecular Devices). The data was plotted as velocity vs. substrate using GraFit version 7 (Erithacus Software) and the V_max_ and K_m_ of the wild-type and each mutant mPlm were determined. The catalytic efficiency (Kcat/Km) was calculated according to the active enzyme concentration. For accessing the non-specific cleavage during activation, about 10 μg of wild-type and mutant mPlg were activated with different concentrations of (around 0.03 μg) of SAK at various temperature for different times, run on a 4-12% BT gel, and stained with Coomassie Blue. For N-terminal sequencing of the cleaved bands, the activated wild type mPlg and mutants were run on a 4-12% BT gel, transferred to a PVDF membrane, and then stained with Coomassie Blue. Desired bands were cut out from the membrane and sent for N-terminal sequencing using Edman Degradation at Iowa State University Protein Facility.

### Fibrinolysis assay

A classical method was used for fibrinolytic assay [19,20]. Briefly, fibrin plates (9-cm BD Falcon Petri dish) were made by combining fibrinogen (1.2 mg/ml final concentration) and thrombin (0.3 NIH units/ml final concentration) in a 1.4% agarose solution. About 0.3-cm diameter/0.22-cm height holes were punched after the agarose plates solidified. Activated mPlg samples (15 μl) were added to the holes to start the fibrinolysis reactions, after which the plates were incubated at 37 for 5 and 10 hours. At each time, the areas of the digest cycles were measured and photograph were taken.

### Staphylokinase (SAK) expression and purification

A synthetic SAK gene was constructed with optimized *E. coli* codons according to protein sequence (accession number: CAA24957 [21]). The synthetic gene was then cloned into a T7 expression vector and the *E. coli* expressed SAK was purified using ion exchange chromatography. The activity of purified SAK was accessed using the Plg activation method described above.

## Results

We performed studies in a canine model which showed our recombinant wild-type mPlm dissolved artificially induced blood clots better than tPA (results not shown). In a separate collaboration, a more detailed study of mPlm in a canine artificial PAO model showed that mPlm is better in reperfusion than tPA: while none of the animals in mPlm group experienced reocclusion after treatment, 20% of the tPA group reoccluded [22]. These results clearly demonstrate the effectiveness of mPlm in treating PAO in the animal models.

**Figure 1:**
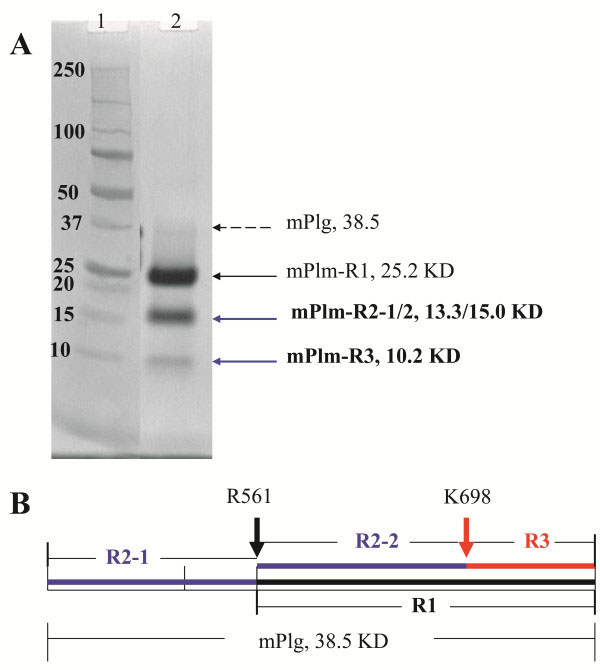
**Cleavage of mPlg activated by uPA. A.** SDS-PAGE showing the products of purified mPlg (38.5 KD) after being activated using uPA. The same results were obtained when SAK was used. Lane 1 is a molecular weight marker and Lane 2 is the activated mPlm. **B.** Diagram of fragments R1, R2-1, R2-2, and R3. The left arrow shows the authentic activation cleavage between R^561^ and V^562^. This cleavage results in normal activation fragments R1 (25.2 KD) and R2-1 (13.3 KD). The right arrow shows the non-specific cleavage between K^698^ and E^699^, which results in extra-fragments of R2-2 (15.0 KD) and R3 (10.2 KD).

**Figure 2:**
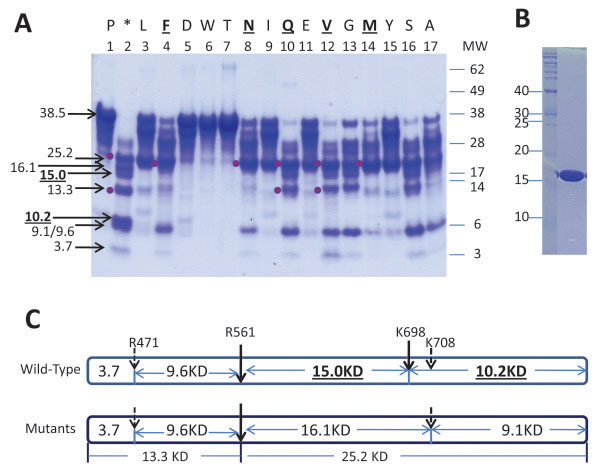
**Activation sites of wild-type and mutant mPlg. A.** SDS-PAGE of wild-type and mutant mPlm activated with SAK. Standard molecular weight is indicated on the right side. The number system for each mutant and the wild-type is labeled at the top, and is the same for Table 2. In addition, mutations for each lane are labeled at the top of the figure with single amino acid letters. The two bands (15.0 and 10.2 KD) from the major non-specific activation site at position K698 of the wild-type enzyme are highlighted at the left side of the figure. These two bands are not present in mutant lanes. After activation at the R561 site (shown in C) for both wild-type and the mutants, two major bands (25.2 and 13.3 KD) appear and are indicated with dots at the left side of the band. Molecular weights of other minor, non-specific cleavage bands are also labeled at the left. **B.** Purified SAK (16 KD) used in this experiment. **C.** Schematic presentation of the cleavage sites derived from A. The activation site at R561 is shown for both the wild-type and the mutants. The top scheme shows the cleavage pattern for the wild-type mPlm, which includes a major non-specific cleavage site at K698, and two of the minor non-specific cleavage sites. The bottom scheme shows the cleavage pattern for the mutants, in which the K698 position is no longer cleavable.

**Figure 3:**
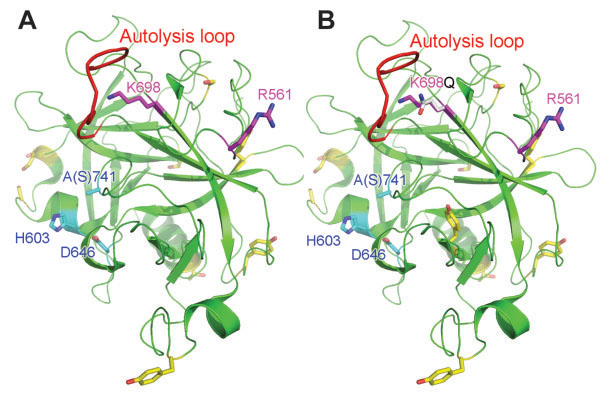
**Autolysis loop, K698 localization, and predicted phosphorylation sites in the structure of μPlg. **The structure is viewed with PyMOL software. The backbone is presented as a ribbon diagram and the Autolysis Loop is colored in red. Side chain colors follows: K698 (purple), R561 (purple), active side triad (H603, D646, S741) (cyan), and the predicted mammalian phosphorylation sites (yellow). The active site serine (S741) was replaced with Alanine (A741) in the structure. **A.** Wild type μPlm. **B.** A model of K698Q mutant. The side chain for the Q residue is in silver color.

During the scale up manufacturing process for mPlm drug development, a problem of serious non-specific cleavage of the mPlm was encountered during the activation process using either uPA (Figure 1A) or SAK (Figure 2A). After amino acid sequencing of the blotted bands using N-terminal Edman degradation we found that the major non-specific cleavage site is between K^698^ and E^699^. A schematic presentation of the resulting fragments is shown in Figure 1B. As shown in the 3-dimentional structure (Figure 3A) [23], the normal activation residue R^561^ is at the activation loop. Cleavage at this site results in normal activation fragments “R1” and “R2-1” shown in Figure 1. The “R1” fragment was further cut at a non-specific site between K^698^-E^699^, resulting in the “R3” and “R2-2” fragments shown in Figure 1. The 3-D structure in Figure 3A shows that K^698^ is located at the C-end of the Autolysis Loop (the same position is Q in human Trypsinogen and Bovine Chymotrypsin). Because trypsin-like serine proteases have rather restricted requirements for basic residues (R, K, H) at their P1 substrate position [24], changing K^698^ residue to amino acids other than arginine and histidine should eliminate the non-specific cleavage. Cysteine residue was also avoided in the mutagenesis studies because of possible complications from oxidation and incorrect disulfide bond formation during refolding. Because of the difficulties inherent in calculating a specific change that may result in better refolding, stability, and activity, we decided to use a saturation mutagenesis strategy to change K^698^ to the rest of the 16 amino acid residues, and to select the best mutant for further drug development studies.

**Figure 4:**
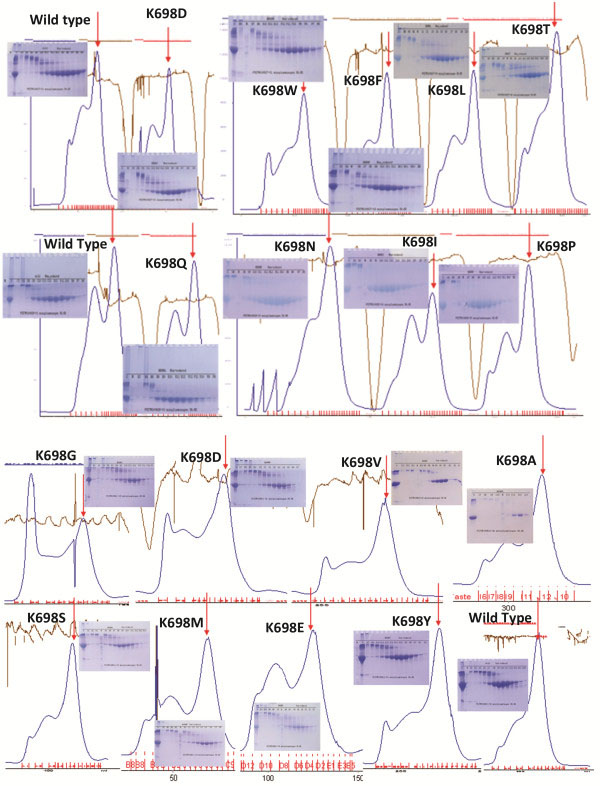
**Purification of mutant mPlg using Superdex-200 SEC column. **Results of wild type and all 16 mutants are shown. Each mutant was expressed, refolded, and purified as described in the methods section. Non-reduced SDS-PAGE (4-12% polyacrylamide gradient gel) was used to access the folding. Each batch of refolding was performed with the wild-type enzyme as a positive control. On the SDS-PAGE, the lower major band in the second peak is the folded enzyme. In the SEC graph, the folded, active peak is indicated with red arrows.

We constructed a mutant for each amino acid change and cloned all 16 mutant expression vectors. The expression levels of all mutant mPlg in *E. coli* are similar to that of the wild-type’s. The IBs were purified and refolded in the same way as the wild-type. The refolded samples were concentrated by ultrafiltration, and purified with Superdex 200 column chromatography (Figure 4), as described in our previous publication [15]. In each SEC graph shown in Figure 4, the first peak eluted from the void volume is the non-folded soluble aggregates, and the latter peak eluted, indicated by an arrow, is the folded, active protein. The non-reduced SDS-PAGE of each column fraction in the insert shows the first aggregation peak and the second folded peak at the predicted molecular weight of the mPlg monomer. As shown in Figure 4, all of the designed K^698^ mutants can be expressed, refolded, and purified with similar characteristics as that of the wild-type mPlg. In order to ensure that results are comparable and reliable, we purified IBs of each batch of mutants (6-10) along with the wild-type zymogen, and refolded them at the same time. The purified and folded fractions were used for activity tests and activation studies.

The main purpose of this study is to eliminate the non-specific cleavage site at the K^698^ position. Figure 2A shows the non-specific cleavage bands (15.0 and 10.2 KD) at Lane 2 of the wild-type enzyme. These two bands are not present in any lanes of the mutants, indicating that the non-specific cleavage at K^698^ was eliminated in the mutants, validating the original goal of the design (Figure 2A). The homogeneity of the SAK used in the activation process is shown in Figure 2B, indicating that SAK was highly purified. N-terminal amino acid sequencing of the cleavage fragments revealed other minor non-specific cleavage sites under some experimental conditions, which were designed to “over digest” in order to clearly identify the non-specific cleavage sites, corresponding to molecular weight 16.7, 8.5, and 4.8 kDa. Two of the minor cleavage sites, along with the activation site and the K^698^ non-specific cleavage site, are depicted in Figure 2C. The top of Figure 2C depicts the activation cleavage sites of the wild-type mPlg: the activation site at R^561^, the major non-specific cleavage site at K^698^, and two of the other minor non-specific cleavage sites. The bottom of Figure 2C depicts the activation cleavage sites of the mutant mPlg: the same activation site at R561, and two of the other minor cleavage sites under certain experimental conditions. The K^698^ cleavage site, however, is completely eliminated from all of the mutant mPlg studied.

The purified wild-type and mutant mPlg were activated with SAK, and kinetic parameters were measured and compared as shown in Table 2. As expected, most of the mutants have a lower refolding or catalytic efficiency relative to wild-type. Of the 16 mutants, five have higher refolding and catalytic efficiencies than other mutants, although the Kcat/Km value are still lower than the wild-type. These mutants, K698F, K698N, K698Q, K698V, and K698M, along with the wild-type enzyme, are highlighted with bold letters in Table 2. In particular, the active mutants K698F, K698N, and K698Q have significantly higher catalytic efficiency than the other mutants.

**Table 2:** Kinetic parameters of wild-type and mutant mPlg*

**mPlm**	**Active μM in 0.5 μM**	**Vmax (μM/Min)**	**Km (μM)**	**Kcat (Min **^**-1**^**)**	**Kcat/Km**	**Kcat/Km Ratio**
					**(μM**^**-1 **^**Min**^**-1**^**)**	
01_K698P	0.00375	11 ± 0.5	2145.3 ± 145.5	2933	1.37	0.29
**02_WT**	**0.1265**	**207 ± 4.9**	**347 ± 25**	**1636**	**4.72**	**1**
03_K698L	0.0685	126 ± 6.8	2459 ± 201	1839	0.75	0.16
**04_K698F**	**0.124**	**97 ± 4.6**	**439 ± 59**	**782**	**1.78**	**0.38**
05_K698D	0.046	17 ± 1	587 ± 83	370	0.63	0.13
06_K698W	0.0065	72 ± 8	9686 ± 1222	11077	1.14	0.24
07_K698T	0.007	24 ± 1	4107 ± 221	3429	0.83	0.18
**08_K698N**	**0.1625**	**224 ± 4.4**	**811 ± 35**	**1378**	**1.7**	**0.36**
09_K698I	0.399	152 ± 6.6	1587 ± 118	381	0.24	0.05
**10_K698Q**	**0.129**	**213 ± 6.5**	**1406 ± 76**	**1651**	**1.17**	**0.25**
11_K698E	0.1905	62 ± 2.8	2411 ± 162	325	0.13	0.03
**12_K698V**	**0.154**	**202 ± 5.6**	**1609 ± 77**	**1312**	**0.82**	**0.17**
13_K698G	0.108	11 ± 1.4	617 ± 181	102	0.17	0.03
**14_K698M**	**0.127**	**221 ± 5.6**	**1664 ± 71**	**1740**	**1.05**	**0.22**
15_K698Y	0.3545	33 ± 1.9	2451 ± 209	93	0.04	0.01
16_K698S	0.1405	39 ± 1.3	1776 ± 96	278	0.16	0.03
17_K698A	0.046	11 ± 0.7	885 ± 111	239	0.27	0.06

*Column 1 in Table 2 shows different mutants (WT: wild-type). Column 2 shows the active enzyme calculated from active site titration, which is an indication of either refolding efficiency or stability of the active zymogen. The concentration of the active enzyme was used to calculate Kcat. Columns 3-7 show the measured and calculated kinetic parameters; column 7 shows the ratio of the catalytic efficiency of different mutants compared with the wild-type, which is set to 1.

We tested the activity of selected mutants by fibrin hydrolysis, in comparison with the wild-type enzyme (Table 3). Although the table shows that the hydrolysis rates of the two mutants (K698N, K698Q) are significantly lower than that of the wild-type’s, further research may prove the “druggability” of the selected mutants toward thromboembolism related diseases, as will be discussed below.

**Table 3:** Fibrinolytic activity of wild-type (WT) and mutant mPlm

**conc. (mg/mL)**	**Digestion area (mm**^**2**^)*		
	**WT**	**K698N**	**K698Q**
0.05	125.7 ± 4.2	126.7 ± 8.5	71.1 ± 10.6
0.1	194.9 ± 8.8	151.8 ± 9.3	100.8 ± 20.1
0.2	217.9 ± 16.6	189.9 ± 1.7	138.0 ± 13.2
0.4	279.1 ± 2.1	233.8 ± 9.6	182.9 ± 18.6
0.8	308.1 ± 22.0	268.8 ± 4.1	203.7 ± 10.7

*Area at each data point was calculated (*A* = *πr*^2^) by measuring the radius ® of the digestion ring.

## Discussion

Our original interest in mPlm stemmed from results of the animal studies cited above [25-27], experience in the refolding of μPlg and mPlg, and structural interaction with the PA streptokinase (SK) [23,28,29]. Reports in the literature used 2.5-5.0 mg/kg μPlm to treat PAO in a rabbit model [27], and similar amounts of mPlm in a canine model [22]. Extrapolating to humans, an infusion dose of as much as 200 mg would be required in a typical treatment (correcting for interspecies differences between animals and humans). Because of the large quantities of mPlm required for therapeutic efficacy, using a mammalian, insect, or yeast (only ~3 mg/L, but with no reports of functionality [30]) system to express the protein for clinical development is not only cost-prohibitive but also technically challenging. In contrast to the monumental challenges involved in production and expression in eukaryotic systems, expression in *E. coli* is optimally suited for this task. However, mPlm can only be over-expressed in the insoluble inclusion body form in *E. coli* (laboratory experience). We are able to routinely obtain >1.2 g/L >80% pure mPlg in the form of inclusion bodies from expression in shaker flasks. The yield can potentially be much higher if an optimal procedure is established in a fermentation system. Thus expression in the inactive inclusion body form will be an effective way to obtain large quantities of the protein for thrombolytic drug development.

As described, despite success in mPlg refolding, purification, and functional studies (including animal tests), we encountered a serious non-specific cleavage problem during activation using either uPA or SAK (Figure 1, 2). We have also observed autoactivation problems during long-term storage at 4^°^C [15], which is preventable in our experience by controlling storage buffer composition and storage temperature (frozen versus 4^°^C). On the other hand, our results have shown that the non-specific cleavage problem during activation could not be avoided by controlling the activation conditions. We have tested many different activation conditions including various buffer conditions, different temperatures, and various activation times but the non-specific cleavage problem could not be eliminated. Non-specific cleavage during activation decreases the specific activity of the purified mPlm, rendering clinical development difficult if not impossible. As a first step towards solving this problem, we identified the non-specific activation site by N-terminal amino acid sequencing. Because Plg purified from human serum can be activated to Plm without non-specific cleavage, there must be structural reasons for the non-specific cleavage at a specific site (K^698^) of the recombinant mPlg. We postulate three possible mechanisms for the non-specific cleavage. The first mechanism is that a certain percentage of the purified mPlg was not refolded into the native conformation, but rather in an inactive conformation that can easily be cleaved at position K^698^. From our results shown in Table 2, under the experimental condition described, when the wild-type mPlg was activated without further purification, only about 25% is in active form. The inactive form might be in conformations such that the K^698^ position is readily accessible for cleavage. The second possible mechanism is that, under the activation conditions, the refolded protein may be in a “looser” conformation with respect to the native conformation of the wild type enzyme, and therefore may be more susceptible to non-specific cleavage. We have previously experienced similar situations in which certain folded proteins are less stable than those that are natively expressed. One of the main reasons for the “loose” conformation may be the differences in the post-translational modifications between mammalian and bacterial hosts. This leads us to the third possible mechanism of non-specific cleavage, which is the post-translational modification difference. The post-translational modifications that may influence the stability of mPlg include glycosylation, phosphorylation, and sulfation. Using the ExPASy server (http://www.expasy.org/), we identified no predicted glycosylation site in mPlg. However, we have identified 18 mammalian phosphorylation sites in mPlg: 8 Ser sites, 4 Thr sites, and 6 Tyr sites. These sites are labeled in the 3-D structure of μPlg (Figure 3), which clearly shows that almost all of the predicted phosphorylation residues are located on the surface of the protein (with the majority on surface loops). Besides possible regulatory roles, phosphorylation may contribute to the solubility and stability of the native protein. Although there are specific studies for protein phosphorylation in *E. coli*[31], methods to predict the specific phosphorylation of mammalian proteins expressed in *E. coli* remain elusive. On the other hand, there is strong evidence that phosphorylation patterns in *E. coli* differ from those in mammalian cells, and this may contribute to the “loose” conformation of the refolded mPlg under the activation conditions when expressed in *E. coli*. For other modifications, there is only one predicted sulfation site in mPlg (Y^535^), which is located at the kringle region and is unlikely to influence the refolding and stability of the catalytic domain of mPlg. For therapeutic drug development, using peggylation may reduce the possible immunogenesis problems of the refolded protein, with possible trade off of reduced activity.

We performed preliminary kinetic measurements of the mutants as comparing with the wild-type mPlm, as shown in Table 2. Since this is the first screening step toward our ultimate goal of therapeutic drug development, we did not optimize refolding and purification conditions for the mutants. It is likely that each mutant will require different optimized refolding and purification conditions, and the conditions we optimized for the wild-type may not be suitable for the mutants. Although it is possible that some mutants are inherently less efficient than the wild-type, we believe that the lack of optimization may be the major reason that the catalytic efficiency of the selected mutants (e.g. K698N and K698Q) are significantly lower than the wild-type’s, as shown in Table 2 and Table 3. As the next step of our planned drug development program, we will be concentrated on optimize the refolding and purification conditions for K698N and K698Q, and ultimately select one mutant as our therapeutic drug candidate.

## Conclusion

In this study, we used structure based mutagenesis and screening methods to select protein therapeutics that are more amendable for the drug manufacturing procedure development. Figure 1 clearly shows K^698^ cleavage fragments, and Figure 2 shows additional “minor” cleavage sites at the R^474^ and R^814^ positions. The differences between these two results are procedural: in Figure 1, conditions were controlled to achieve complete activation of the wild-type enzyme without “overkill” to produce minor cleavage. The activated product was further purified before running the SDS-PAGE. In Figure 2, in order to observe the differences between the wild-type and mutants, we over digested the zymogen and used crude digests to run the SDS-PAGE. The minor cleavage sites shown in Figure 2 were therefore unavoidable.

In addition, Table 2, column 2 shows an active site titration of the wild-type and mutant mPlm. The titration shows that under the activation conditions, only about 25% of the wild-type mPlm is active, with some mutants active and the rest in soluble, inactive forms. The inactive proteins may have incorrectly folded conformations or they may have lost activity because of over digestion. Table 2 also shows that the active fractions of most of the mutants are lower than the wild-type enzyme, with exceptions of K698I, K698Y, K698N, and K698E. The results could be because most of the mutants are less stable than the wild-type, but could also be due to the fact that the refolding, purification, and activation conditions were not optimized for each mutant under the experimental conditions. Some of the mutants, such as K698P, K698W, and K698T have approximately 50-fold lower active form and 10-fold higher Km values when compared to wild type, indicating they are inactive mutants. These mutants are difficult to refold and therefore are not suitable for drug development. As shown in the Results section, K698F, K698N, and K698Q have significantly higher catalytic efficiency than the other mutants. In addition, the fibrinolytic assay shows that although the catalytic efficiency of the selected mutants is slightly lower, for practical applications the efficacy can be compensated by applying higher amounts of the selected mutants (Table 3). Modeling of the 3-demensional structure shows that replacing K698 with glutamine (K698Q) results in minimal structural disturbance (Figure 3B). The side-chain of K698Q has similar length relative to the wild-type K698, and both have a soluble terminal residue in which the only difference is the charge at neutral pH in a water solution. Taking into account the data from Figure 2 and Table 2, as well as structural considerations shown in Figure 3B, we identified K698N and K698Q as our lead candidate for further drug development.

## Competing interests

The authors declare that they have no competing interests.

## Authors’ contributions

XLL supervised the study design, interpreting of results, manuscript preparation and wrote the manuscript. XTL, YW, YWW, and BH performed the cloning, mutagenesis, expression, refolding, purification, activity studies, assist in acquiring and analyzing some of the data and helped write the manuscript. JJL and SJH performed some of the kinetic studies, assist in acquiring and analyzing some of the data, and helped write the manuscript. HY, HWS, and JY performed the animal studies, assist in acquiring and analyzing some of the data and helped write the manuscript. BH and XCZ performed the structural analysis and design and helped write the manuscript. WC and XMX helped the study design and directed some of the other authors in experimental details, interpreting of results, assist in acquiring and analyzing some of the data and helped write the manuscript. All authors read and approved the final manuscript.
